# Factors associated with tuberculosis by HIV status in the Brazilian national surveillance system: a cross sectional study

**DOI:** 10.1186/1471-2334-14-415

**Published:** 2014-07-28

**Authors:** Thiago Nascimento do Prado, Angélica Espinosa Miranda, Fernanda Mattos de Souza, Elias dos Santos Dias, Lorena Kellen Fernandes Sousa, Denise Arakaki-Sanchez, Mauro N Sanchez, Jonathan E Golub, Ethel Leonor Maciel

**Affiliations:** Lab-Epi UFES – Laboratório de Epidemiologia, Universidade Federal do Espírito Santo, Av. Marechal Campos, 1468- Maruípe, Vitória, ES Brazil; School of Nursing, Federal University of Espírito Santo, Espírito Santo, Brazil; Post-Graduate Program in Infectious Diseases, Federal University of Espírito Santo, Espírito Santo, Brazil; Post-Graduate Program in Public Health, Federal University of Espírito Santo, Espírito Santo, Brazil; TB National Consultant, Pan American Health Organization, São Paulo, Brazil; Department of Public Health, University of Brasilia, Federal District, Brazil; Center for Tuberculosis Research, Johns Hopkins University School of Medicine, Baltimore, MD USA

**Keywords:** Tuberculosis, HIV, Coinfection, Logistic regression

## Abstract

**Background:**

Over the last decade tuberculosis (TB) incidence and mortality in Brazil have been steadily declining. However, this downward trend has not been observed among HIV-infected patients. We describe the epidemiological and clinical profile of TB patients by HIV status using the Brazilian National Surveillance System.

**Methods:**

All TB diagnoses with HIV status information between January 1, 2007 and December 31, 2011 were categorized as either HIV or non-HIV at time of TB diagnosis. Co-infected patients (TB-HIV) were compared to TB patients with no HIV-infection using a hierarchical logistic regression model using Stata 13.0.

**Results:**

The prevalence of TB-HIV co-infection was 19% among adults ≥ 15 years of age. We analyzed data from 243,676 individuals, of whom 46,466 were TB-HIV and 197,210 were only TB cases. The following factors increased risk of co-infection: male sex (OR: 1.06, 95% CI 1.03-1.10), 20 to 39 years of age (OR = 4.82, 95% CI 4.34-5.36), black (OR = 1.08, 95% CI 1.04-1.13), 4–7 years of education (OR = 1.13, 95% CI 1.19-1.28), diagnosed following default (OR = 2.65, 95% CI 1.13-6.25), presenting with pulmonary and extra-pulmonary forms of TB simultaneously (OR = 2.80, 95% CI 1.56-5.02), presenting with histopathologic examination suggestive of TB (OR = 2.15, 95% CI 1.13-4.07). Co-infected patients were less likely to live in rural areas (OR = 0.45, 95% CI 0.42-0.48), have diabetes (OR = 0.45, 95% CI 0.40-0.50) and be smear positive (OR = 0.55, 95% CI 0.32-0.95), and co-infected patients had higher risk of default (OR = 2.96, 95% CI 2.36-3.71) and death from TB (OR = 5.16, 95% CI 43.04-5.77).

**Conclusions:**

The prevalence of co-infection with HIV among TB patients is 19% in Brazil. By identifying predictors of co-infection targeted interventions can be developed to prevent both TB and HIV, and to diagnose each disease earlier and ultimately decrease poor treatment outcomes and death.

**Electronic supplementary material:**

The online version of this article (doi:10.1186/1471-2334-14-415) contains supplementary material, which is available to authorized users.

## Background

Tuberculosis (TB) remains a serious public health problem, especially in developing countries like Brazil, which is one of the 22 high burden countries of the disease worldwide [1]. Over the last decade TB incidence and mortality in Brazil have been steadily declining. However, this downward trend has not been observed among HIV-infected patients [2, 3].

Since the 1980s, HIV has been one of the main factors contributing to the resurgence of TB in developed and developing countries alike [4]. The virus has changed the natural history of active tuberculosis as well as having a marked impact on the epidemiology and clinical outcomes of TB [5, 6]. HIV infected patients have an annual risk of reactivating latent TB infection between 3 and 15% compared to 0.01 to 0.1% for the general population [7].

The implementation of collaborative TB-HIV activities is still modest in Brazil. These activities include coordination between the HIV/AIDS and TB programs for delivering integrated TB and HIV services including testing TB patients for HIV, providing ART to TB co-infected patients, providing HIV prevention services for TB patients, intensifying TB case finding among people living with HIV, and offering isoniazid preventive therapy. Tuberculosis in HIV-infected patients is often not identified until death, highlighting a failure of the health system to detect both diseases earlier [8]. A recent study of the six Brazilian states with the highest reported levels of HIV data in the Brazilian national TB reporting system, reported that 40% of TB patients had no HIV status provided [9].

Studies conducted in Brazil and other high burden TB countries have reported several socio-demographic and clinical features significantly associated with TB-HIV co-infection [10–12]. However, to the authors’ knowledge, there has not been such a study done in Brazil using the Epidemiological Surveillance System. In addition, none of these studies used models of analysis using the hierarchical multivariable analysis, which can detect the factors associated with TB-HIV co-infection relative to TB only in Brazil.

In this study, we describe the epidemiological and clinical profile of TB patients by HIV status using the Brazilian National Surveillance System.

## Methods

### Study design

This is a cross sectional study utilizing the database of the national TB reporting system (SINAN/TB). SINAN was developed in the early 90s, with the objective of collecting and processing data on disease notification throughout the country. SINAN is the primary information system from which data are extracted for epidemiological analyses [13]. This system is available in the website http://dtr2004.saude.gov.br/sinanweb[14]. Although for this particular study, data were obtained from the Tuberculosis National Program at the Ministry of Health in order to avoid replication and misclassification. These databases support the improvement of health care systems in Brazil by increasing the capacity of health care workers to make decisions based on accurate information.

### Study population

The population of the study included TB cases aged ≥ 15 years reported in Brazil between January 1 2007 and December 31, 2011.

### Variables, data collection

The following socio-demographic covariates were evaluated: age (<20 years, 20–39 years, 40–59 years and ≥ 60 years), gender (male, female), skin color (white, black, mixed and other (Asian and indigenous), school level (<4 years, 4 to 7 years, ≥ 8 years), area of residence (urban, rural or peri-urban) and whether the individual was institutionalized (no or yes). The presence of diabetes and alcoholism was included.

The covariates related to TB included the type of TB diagnosed during the study period, classified as 1) new TB case (no prior TB diagnoses), relapse (completed a previous TB treatment) or, 3) return after default (individuals that defaulted from a previous TB treatment regimen and returned to continue treatment). We also included site of TB at presentation (pulmonary, extra pulmonary, pulmonary + extra pulmonary), localization of extra-pulmonary TB, tuberculin skin test (positive if higher than 10 mm), existence of chest X-ray suspicious for TB, result of initial sputum smear test, result of initial culture examination, and result of initial histopathologic examination. Receiving directly observed therapy (DOT) was also included as a covariate. Final treatment outcome was classified as cured (completed treatment and had at least two negative results of smear examination), default (those that did not attend to regular appointments for more than 30 days), TB death, other cause of death (died during TB treatment of another cause), transferred or developed MDR TB.

### Data analysis and statistics

We compared individuals with TB and HIV (TB–HIV) with those who only had TB (TB only) according to socio-demographic and clinical characteristics. Pearson chi-square test was used to compare proportions. Covariates associated (p ≤ 0.05) with the outcome of interest were included in a hierarchical logistic regression model.

Tuberculosis is a disease with a complex causal chain. Constructing a hierarchical model may be a better way to capture the interrelationships between its determinants. In this model, variables are included from distal to proximal ones, according to different levels of a causal network arising from a robust theoretical base [15, 16]. Associations resulting from the hierarchical regression model are adjusted for the variables in the same level and those in previous levels, taking into account both confounders and mediators [15, 16].The present model was based on the conceptual framework for social determinants of TB formulated by Maciel [17]. In the hierarchical analysis, the following covariates were included: step 1 (Gender + age + school level + skin color + Area of residence); step 2 (variables retained from step 1+ Institutionalization + DOT); step 3 (variables retained from step 2 Diabetes); step 4 (variables retained from step 3 + treatment type _+_ TB form + Tuberculin Skin Test + smear + culture + pathologic examination + X ray suspicious for TB); and step 5 (variables retained from step 4 + outcome). In each step, those covariates associated with the outcome (p ≤ 0.05) were retained in the model. These analyses were conducted with Stata, version 13.0.

Due to the high proportion of missing information on HIV status, we carried out polynomial analysis based on the following TB treatment outcomes (cure, default, death from TB, death from other causes and MDR TB) comparing to HIV status (negative, positive, HIV test was requested and test not done). Cured TB status was used as a reference category instead of the present rendition.

### Ethics statement

The databases were obtained under the rules for release of the Secretariat of Health Surveillance and Health Care Department of the Ministry of Health, ensuring the confidentiality and nondisclosure of individual identifiers. The Federal University of Espirito Santo (UFES) Institutional Review Board approved the study design by registration number 466/12.

## Results

**Figure 1:**
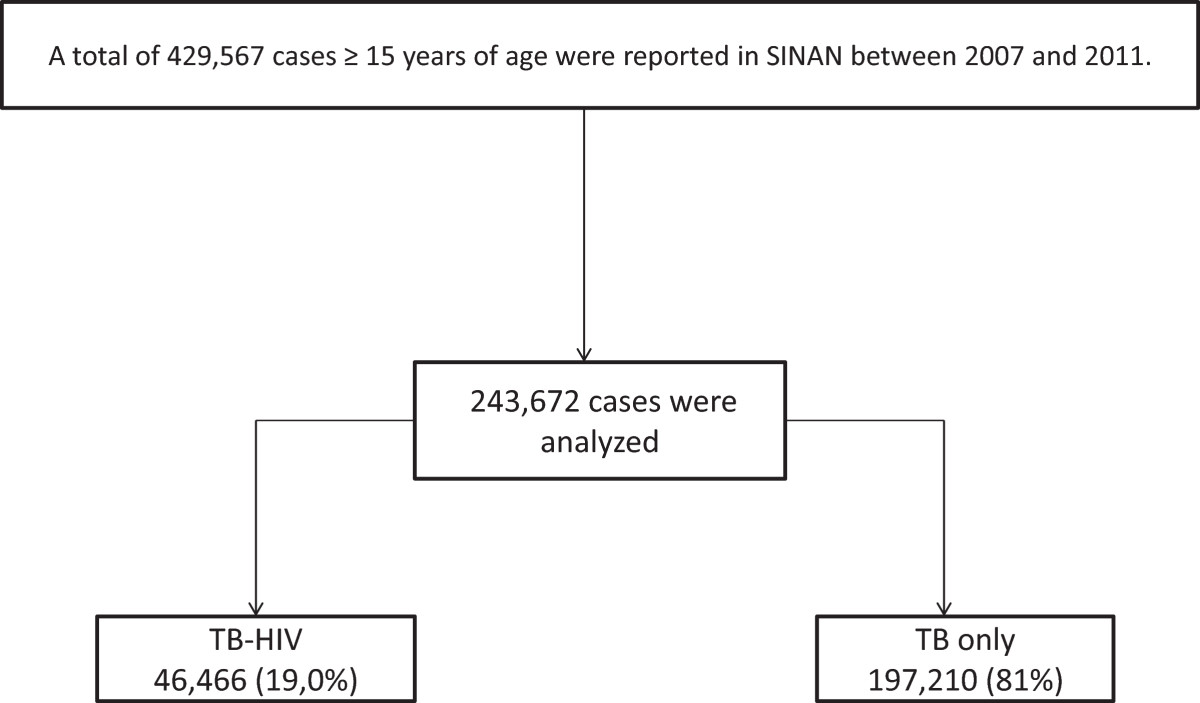
**Study Flow Diagram; TB-HIV: Tuberculosis and HIV co-infection subjects; TB only: subjects who had only tuberculosis diseases.**

Between 2007 and 2011, 429,567 adult cases of TB were reported in SINAN, of which 185,891 (43.27%) were missing data for HIV status. Among 243,676 TB patients with known HIV status, 46,466 (19%) were TB-HIV co-infected (Figure 1).

Individuals self-identified as black were more prevalent in the TB-HIV group (15.88%) compared to the TB only group (12.81%), (p < 0.001) (Table 1). The proportion of subjects with < 4 years of education was higher in the TB only group (42.4%) than TB-HIV group (38.0%), (p < 0.001). Most individuals lived in urban areas, though the proportion was slightly higher in the TB-HIV group (95.57% vs 90.56%) (p < 0.001). Diabetes was less prevalent in TB–HIV group (2.65%) compared to the TB only group (6.77%) (p < 0.001). Alcoholism was similar between groups (17.40% vs 16.94%; p = 0.027).

**Table 1:** **Distribution of socio-demographic characteristics of tuberculosis (TB) cases according to HIV status in Brazil, 2007-2011**

Characteristics (N*)	TB-HIV	TB only	p**
	n (%)	n (%)	
Gender (243,670)			
Female	13,899 (29.91)	64,862 (32.89)	< 0,001
Male	32,567 (70.09)	132,342 (67.11)	
Age (243,676)			
<20 years	715 (1.54)	12,042 (6.11)	< 0,001
20-39 years	26,610 (57.27)	94,448 (47.89)	
40-59 years	17,554 (37.78)	67,495 (34.22)	
≥60 years	1,587 (3.42)	23,225 (11.78)	
Skin color (225,264)			
White	19,919 (47.10)	86,109 (47.06)	< 0,001
Black	6,716 (15.88)	23,432 (12.81)	
Browns	15,253 (36.06)	70,377 (38.46)	
Other	406 (0.96)	3,052 (1.67)	
School level (123,482)			
<4 years	8,970 (38.06)	42,378 (42.41)	< 0,001
4-7 years	7,418 (31.48)	24,672 (24.69)	
≥ 8 years	7,179 (30.46)	32,865 (32.89)	
Area of residence (165,113)			
Urban	32,049 (95.57)	119,150 (90.56)	< 0,001
Rural	1,301 (3.88)	11,406 (8.67)	
Periurban	186 (0.55)	1,021 (0.78)	
Institutionalization*** (202,340)			
No	35,878 (92.63)	152,237 (93.05)	0.004
Yes	2,853 (7.37)	11,372 (6.95)	
Diabetes (220,039)			
No	40,213 (97.35)	166,625 (93.23)	< 0,001
Yes	1,095 (2.65)	12,106 (6.77)	
Alcoholism (221,868)			
No	34,161 (82.60)	149,930 (83.06)	0,027
Yes	7,194 (17.40)	30,583 (16.94)	

*Number of valid observations. **Pearson’s chi-squared test. ***prisoners.

Table 2 describes the individuals according to characteristics of TB presentation. New TB cases were reported more among the TB only group (83.89%) than the TB-HIV group (73.77%, p < 0.001). A greater proportion of the TB group (62.41%) had a recorded positive tuberculin skin test compared to the TB-HIV group (41.17%; p < 0.001). Prevalence of both pulmonary and extrapulmonary TB in the same individual was more prevalent in the TB – HIV group (11.90% vs 2.68%, p < 0.001). An X-ray suggestive for TB was found in 86.60% of the TB–HIV group and 94.0% of the TB only group (p < 0.001). On the other hand, smear and culture positivity were more prevalent among the TB only group (69.34% vs 52.22%, p < 0.001) and (60.95% vs 59.24%, p < 0.001), respectively (Table 2).

**Table 2:** **Distribution of presentation and treatment characteristics of tuberculosis (TB) cases according to HIV status in Brazil, 2007-2011**

Characteristics (N*)	TB-HIV	TB only	P**
	n (%)	n (%)	
Type of Entry (243,676)			
New case	34,278 (73.77)	165,449 (83.89)	< 0,001
Relapse	4,418 (9.51)	12,235 (6.20)	
Return after default	4,681 (10.07)	10,471 (5.31)	
Unknown	240 (0.52)	245 (0.12)	
Transferred	2,849 (6.13)	8,810 (4.47)	
Tuberculin skin test (40,313)			
Negative	4,841 (58.83)	12,060 (37.59)	< 0,001
Positive	3,388 (41.17)	20,024 (62.41)	
TB form (243,670)			
Pulmonary	29,738 (64.00)	165,978 (84.16)	< 0,001
Extra pulmonary	11,193 (24.09)	25,949 (13.16)	
Pulmonary + Extra pulmonary	5,531 (11.90)	5,281 (2.68)	
X-Ray (208,432)			
Negative	5,212 (13.40)	10,178 (6.00)	< 0,001
Suspicious of TB	33,676 (86.60)	159,366 (94.00)	
Smear (199,235)			
Negative	15,793 (47.78)	50,945 (30.66)	< 0,001
Positive	17,258 (52.22)	115,239 (69.34)	
Culture (60,671)			
Negative	4,421 (40.76)	19,456 (39.05)	< 0,001
Positive	6,425 (59.24)	30,369 (60.95)	
Histopathologic examination (28,618)			
AFB^***^ positive	3,256 (46.91)	6,922 (31.93)	< 0,001
Suggestive	3,217 (46.35)	13,411 (61.87)	
Not suggestive	468 (6.74)	1,344 (61.87)	
DOT^****^ (226,736)			
No	25,644 (61.68)	86,190 (46.55)	< 0,001
Yes	15,935 (38.32)	98,967 (53.45)	
Outcome (215,884)			
Cure	21,404 (55.75)	150,782 (84.95)	< 0,001
Default	7,247 (18.88)	17,474 (9.84)	
Death from TB	2,700 (7.03)	4,296 (2.42)	
Death from other causes	6,835 (17.80	3,895 (2.19)	
MDR-TB	206 (0.54)	1.045 (0.59)	

*Number of valid observations. **Pearson’s chi-squared test. ***AFB: acid-fast bacilli.; ****DOT: Directly Observed Therapy.

The histopathologic examination had a higher proportion of AFB positive in the TB-HIV group compared with the TB only group (46.91% vs 31.93%, p < 0.001). DOT was greater among the TB only group (53.45% vs 38.32%, p < 0.001) and cure was markedly better as well (84.95% vs 55.75%; p < 0.001) (Table 2).

The hierarchical multivariate model (Table 3) showed that individuals aged 20 to 39 years and 40 to 59 years were more likely to be TB–HIV coinfected compared to individuals < 20 years of age (OR = 4.82, 95% CI 4.34-5.36 and OR = 3.67, 95% CI 3.30-4.09). Individuals > 60 years of age did not have higher TB-HIV co-infection compared to those < 20 years of age. The odds were greater for blacks to be TB–HIV (OR = 1.08, 95% CI 1.04-1.13) and for those with school level between 4–7 years (OR = 1.13, 95% CI 1.19-1.28). Living in rural area was protective of being co-infected (OR = 0.44, 95% CI 0.41-0.48), as well as having diabetes (OR = 0.45, 95% CI 0.40-0.50).

**Table 3:** Hierarchical* multivariate analysis of the association of tuberculosis by HIV status

	Characteristics		OR**	95%CI***
	Gender	Female	Ref.	
Level 1		Male	1,06	1.03-1.10
	Age	<20 years	Ref.	
		20-39 years	4,82	4.34-5.36
		40-59 years	3,67	3.30-4.09
		≥60 years	1.04	0.92-1.18
	Race	White	Ref.	
		Black	1.08	1.04-1.13
		Browns	0,77	0.75-0.80
		Other	0,66	0.57-0.77
	Schooling	<4 years	Ref.	
		4-7 years	1.23	1.19-1.28
		≥ 8 years	0,96	0.92-1.00
	Area of residence	Urban	Ref.	
		Rural	0,44	0.41-0.48
Level 2		Periurban	0,66	0.61-0.77
	DOT****	No	Ref.	
		Yes	0.65	0.98-1.12
	Institutionalization	No	Ref.	
		Yes	1.07	1.00-1.15
Level 3	Diabetes	No	Ref.	
		Yes	0.45	0.40-0.50
	Alcoholism	No	Ref.	
		Yes	1.04	0.99-1.09
Level 4	Type of entry	New case	Ref.	
		Relapse	2.23	1.00-4.94
		Return after default	2.65	1.13-6.25
		Unknown	1	-
		Transferred	0.79	0.35-1.81
	Tuberculin skin test	Negative	Ref.	
		Positive	0.73	0.50-1.07
	TB form	Pulmonary	Ref.	
		Extra pulmonary	1.28	0.75-2.20
		Pulmonary + Extra pulmonary	2.80	1.56-5.02
	Smear	Negative	Ref.	
		Positive	0.55	0.32-0.95
	Histopathologic examination	AFB***** positive	Ref.	
		Suggestive	2.15	1.13-4.07
		Not suggestive	0.71	0.37-1.34
	X-Ray	Negative	Ref.	
		Suspicious of TB	0.62	0.35-1.09
Level 5	Outcome	Cure	Ref.	
		Default	2.79	2.45-3.18
		Death from TB	3.64	2.92-4.53
		Death from other causes	7.93	6.66-9.43
		MDR-TB	1.41	0.74-2.68

*The multivariate analyses: step 1 (Gender + age + schooling + race + Area of residence); step 2 (variables retained from step 1 + Institutionalization + DOT); step 3 (variables retained from step 2 + Diabetes); step 4 (variables retained from step 3 + treatment type+ TB form + Tuberculin Skin Test + smear + + culture + histopathologic examination + X ray suspicious for TB); and step 5 (variables retained from step 4 + outcome). ** Adjusted odds ratio. ***CI: confidence interval. ****DOT: Directly Observed Therapy. *****AFB: acid-fast bacilli.

Patients diagnosed with TB after returning after default were more likely to be co-infected (OR = 2.65, 95% CI 1.13-6.25). Both pulmonary and extrapulmonary TB in the same individual were more likely to be reported as TB – HIV (OR = 2.80, 95% CI 1.56-5.02). TB–HIV co-infected patients were less likely to have a positive smear result (OR = 0.55, 95% CI 0.32-0.95), but more likely to have a suggestive histopathologic examination (OR = 2.15, 95% CI 1.13-4.07).

Finally, TB–HIV patients were more likely to get unfavorable results of tuberculosis treatment including default (OR = 2.79, 95% CI 2.45-3.18), death from TB (OR = 3.64, 95% CI 2.92-4.53) and death from other causes (OR = 7.93, 95% CI 6.66-9.43).

Since 43% of subjects with TB were excluded due to missing information of HIV status, we analyzed the treatment outcome (cure, default, death from TB, death from other causes and MDR TB) for HIV status. TB treatment outcomes were best for those who were known to be HIV negative and worst for those known to be HIV positive (data not shown). On the other hand, TB treatment outcomes for those in whom an HIV test was requested but the result was not available were better than those in whom the test was not done. In a multinomial analysis, considering cure as the reference group and comparing with HIV status (negative, positive, test requested but no result recorded and test not done) showed that death from TB presented an odds ratio of 1.48 with HIV positivity (95% CI 1.43-1.53), whereas the default was 1.07 (95% CI 1.04-1.10) Table 4.

**Table 4:** **TB treatment outcomes by HIV status in Brazil, 2007-2011**

Outcome	HIV status			
	Negative	Positive	Test requested but no result recorded	Test not done
	n (%)	n (%)	n (%)	n (%)
**Cure**	150,782 (84.95)	21,4 (55.75)	24,26 (77.83)	91, 31 (73.99)
**Default**	17,474 (9.84)	7,24 (18.88)	4,74 (15.23)	19,22 (15.58)
**Death from TB**	4,296 (2.42)	2,70 (7.03)	978 (3.14)	6,69 (5.42)
Death*	3,895 (2.19)	6,83 (17.80)	1,03 (3.31)	5,75 (4.66)
**MDR-TB**	1,045 (0.59)	206 (0.54)	153 (0.49)	428 (0.35)

*Death from other causes.

## Discussion

The prevalence of TB – HIV co-infection reported by SINAN was 19% among TB patients ≥ 15 years of age with known HIV status. Co-infection was associated with being male, black, low level of education, living in an urban area and being between the ages of 20–59 [4, 10, 18, 19]. These characteristics demonstrate the social characteristics of HIV-TB co-infection. The same epidemiological profile has been described in several studies in Brazilian cities [4, 20, 21]. These findings have been associated with the lifestyle of young adults often associated with lack of awareness of their vulnerability, exposing them to the HIV virus and tuberculosis [22].

Those socio-demographic characteristics strongly influenced the proportion of TB–HIV co-infection. Since the beginning of the new century, the AIDS epidemic in Brazil has reached new population groups and cities where it had not been previously reported, affecting the less privileged social segments in the country [3, 21, 23]. Such populations have historically been plagued with high TB rates; therefore, the introduction of the AIDS epidemic has worsened the TB problem.

Co-infected patients were less likely to have prevalent diabetes in our study. It is important to highlight that TB patients with diabetes have demonstrated worse outcomes of TB treatment. A study carried out in Brazil using the Brazilian national surveillance system (SINAN) demonstrated that TB patients with diabetes were more likely to die of TB [24].

TB/HIV co-infected patients were less likely to be AFB positive than TB patients only which can be attributed to immunodeficiency. This result corroborate with other published studies which should indicate the need to develop more sensitive diagnostic techniques to confirm TB among HIV co-infected such culture. Another tool is a scoring system used to diagnose smear-negative pulmonary TB in children and adolescents, in HIV-infected adults suspected of having smear negative pulmonary TB. A study conducted in Brazil from a cohort of 2,382 HIV-infected adults [25], 1276 were investigated and 128 were diagnosed with pulmonary TB. The scoring system of the Brazilian Ministry of Health for the diagnosis of pulmonary TB in children and adolescents was adapted by the authors of the present study for HIV-infected adults and presented a good capacity for discriminating patients who did not have pulmonary TB, in the studied population.

The treatment of co-infected TB – HIV patients is difficult. Patients with TB require long-term treatment with various medications. For patients with TB-AIDS, adherence with the treatment regimen is difficult due to the extra burden of drug taking, resulting in higher default rate as showed in our study [26–28]. Because of this difficulty and poor adherence to tuberculosis treatment, new strategies for monitoring treatment should be devised, taking into account the characteristics of the co-infection as recommended by Brazilian National Tuberculosis Control. However, in our study the TB – HIV subjects were less likely to be covered under the DOT, although it was not statistically significant. This retention in DOT for TB/HIV co-infected patients was poor in relation to available AIDS and TB treatment models in Brazil. In addition, in some Brazilian cities there is no encouragement for patients on HAART to receive treatment supervision in the community such food vouchers and transportation subsidies.

We also found similar results as other studies [3, 9, 26], indicating that co-infected patients were more likely to have unfavorable treatment outcomes (default and death from TB). The explanation of this unfavorable outcome in our study is not easy. First, CD4 count is related to the severity and clinical presentation of tuberculosis in co-infection, extrapulmonary and disseminated as observed in our study. Second, among co-infected patients, mortality is commonly related to delayed diagnosis of TB because some HIV-infected individuals postpone seeking health care in order to avoid receiving an AIDS diagnosis [10]. As we can see HIV status plays an important role in TB treatment outcome. However, for 43% of patients reported in SINAN during the study period, HIV status was unknown. This information is similar to what was found by a study [9] carried out in Brazil, from 2003 to 2008. That study showed that the group with unknown HIV status showed intermediate outcomes between the groups above (TB only and TB-HIV), suggesting that this group includes some with HIV infection.

We analyzed the epidemiological profile of tuberculosis patients with unknown HIV status with patients with known status in our study period, and we observed that the group with unknown HIV status shared more similar socio-demographic characteristics with the TB-HIV group, although some clinical characteristics of disease were more similar with TB only group as TB form (pulmonary form was more prevalent, data not shown). These facts, coupled with what the study [9] cited above points out, leads us to believe that the group of patients for which we have no known HIV status is a mix of HIV positive and negative individuals. We cannot quantify or identify the direction of the bias our results may have suffered, but as already mentioned, this is a limitation that is inherent to operational research using secondary data such as national disease reporting systems.

Our study has some other limitations. Firstly, the proportion of missing data for some variables was quite significant. Nevertheless, our large sample size still allowed us to maintain high statistical power for all analyses, but not accounting for potential biases. Secondly, we did not have access to information about antiretroviral therapy (ART) history or CD4 count among our group of HIV co-infected patients. Therefore, we did not know if the patients were diagnosed with TB prior to, concomitant with, or after the diagnosis of HIV infection.

## Conclusions

Our analysis provides better understanding of the socio-demographic and clinical differences between HIV and non-HIV related TB in Brazil, providing evidence for developing targeted interventions directed towards reducing both infections in high risk groups. We recommend that the Brazilian National Tuberculosis Control Plan, which currently recommends all patients diagnosed with TB be tested for HIV, be strengthened through better integration and communication between AIDS and TB programs.

## Authors’ original submitted files for images

Below are the links to the authors’ original submitted files for images. Authors’ original file for figure 1
