# Supplementary material for: Factors associated with tuberculosis by HIV status in the Brazilian national surveillance system: a cross sectional study
Source: BMC Infect Dis. 2014 Jul 28;14:415. doi: 10.1186/1471-2334-14-415 (PMC4122782; doi:10.1186/1471-2334-14-415)
Supplement: Supplementary file 1 — Authors’ original file for figure 1 [file 12879_2014_3715_MOESM1_ESM.pdf]

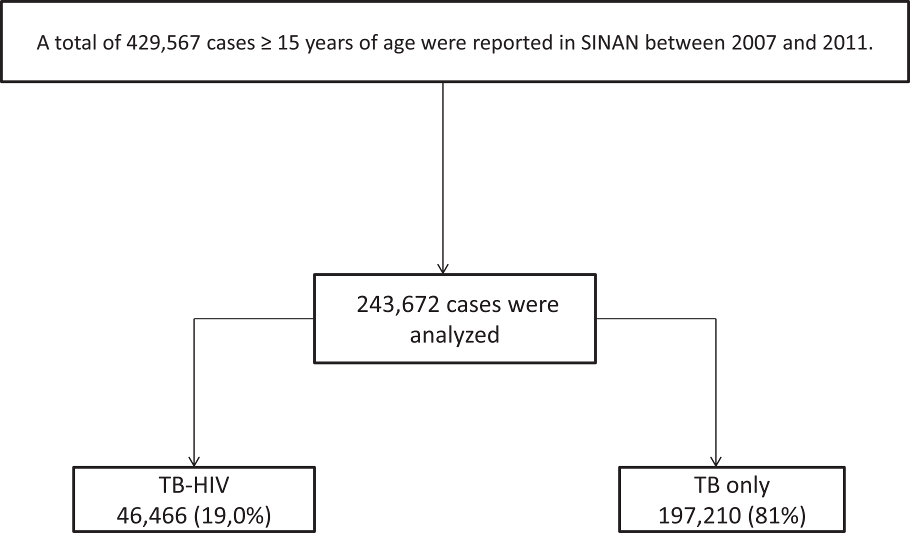
A total of 429,567 cases ≥ 15 years of age were reported in SINAN between 2007 and 2011.
243,672 cases were analyzed
TB-HIV
46,466 (19,0%)
TB only
197,210 (81%)
